# Preimplantation genetic testing for aneuploidy and chromosomal structural rearrangement: A summary of a nationwide study by the Japan Society of Obstetrics and Gynecology

**DOI:** 10.1002/rmb2.12518

**Published:** 2023-05-31

**Authors:** Takeshi Iwasa, Akira Kuwahara, Toshiyuki Takeshita, Yuka Taniguchi, Mikio Mikami, Minoru Irahara

**Affiliations:** ^1^ Department of Obstetrics and Gynecology, Graduate School of Biomedical Sciences Tokushima University Tokushima Japan; ^2^ Clinic Cosmos Kochi Japan; ^3^ Department of Obstetrics and Gynecology Nippon Medical School Tokyo Japan; ^4^ Takeshita Ladies Clinic Tokyo Japan; ^5^ Department of Obstetrics and Gynecology Tokai University School of Medicine Kanagawa Japan

**Keywords:** chromosomal structural rearrangement, PGT‐A, PGT‐SR, recurrent implantation failure, recurrent pregnancy loss

## Abstract

**Purpose:**

The Japan Society of Obstetrics and Gynecology conducted a nationwide clinical study to evaluate the pregnancy outcomes of preimplantation genetic testing for aneuploidy or chromosomal structural rearrangement (PGT‐A/SR).

**Methods:**

Patients that had experienced recurrent implantation failure, recurrent pregnancy loss, or chromosomal structural rearrangement were recruited from 200 fertility centers in Japan. For patients in whom one or more blastocysts were classified as euploid or euploid with suspected mosaicism, a frozen–thawed single embryo transfer (ET) was performed.

**Results:**

A total of 10 602 cycles, maternal age 28–50 years, were enrolled in this study. 42 529 blastocysts were biopsied, and 25.5%, 11.7%, and 61.7% of embryos exhibited euploidy, mosaicism, and aneuploidy, respectively. At least one euploid blastocyst was obtained in 38.3% of egg retrieval cycles with embryo biopsy. A total of 6080 ETs were carried out, and the clinical pregnancy rate per ET, ongoing pregnancy rate per ET, and miscarriage rate per pregnancy were 68.8%, 56.3%, and 10.4%, respectively. The rates of clinical pregnancy and miscarriage remained relatively constant across all maternal ages.

**Conclusions:**

Preimplantation genetic testing for aneuploidy or chromosomal structural rearrangement may improve the pregnancy rate per ET and reduce the miscarriage rate per pregnancy, especially in patients of advanced maternal age.

## 1 INTRODUCTION

Aneuploidy in gametes and embryos is a major cause of implantation failure during in vitro fertilization (IVF) and miscarriage.^1^, ^2^, ^3^ As most aneuploidies arise in maternal meiosis, and they are more common in older women,^4^ the reproductive outcomes of IVF are worse in patients of advanced maternal age. In 2020, the Japan Society of Obstetrics and Gynecology (JSOG) annual online cycle‐based assisted reproductive technology (ART) registry showed that the pregnancy rate and live birth rate per embryo transfer (ET) were 15.8% and 9.9% or lower, and the miscarriage rate per pregnancy was 33.3% or higher in women of 40 years of age or older.^5^ In addition, chromosomal structural rearrangements (CRs), including Robertsonian translocations, reciprocal or balanced translocations, and inversions, are well‐known risk factors for miscarriage. Although morphological assessments are the primary method for embryo prioritization, neither static nor dynamic evaluations can accurately determine chromosome status.

Preimplantation genetic testing for aneuploidy or chromosomal structural rearrangement (PGT‐A/SR) is based on methods for selecting embryos with high potential for implantation and pregnancy and low risk of miscarriage.^6^, ^7^ Initially, cleavage‐stage biopsies and fluorescence in situ hybridization (FISH) were used for PGT‐A/SR, but their efficacy could not be confirmed in randomized control trials (RCT).^8^ Recently, molecular techniques, blastocyst culturing, and embryo vitrification have improved. As a result, they have been extensively utilized for PGT‐A, and some studies have shown that PGT‐A produces favorable pregnancy outcomes per ET in limited infertile populations.^9^, ^10^, ^11^, ^12^ On the other hand, several studies have failed to demonstrate beneficial effects of PGT‐A, even after the improvement of PGT‐A techniques, especially in young populations,^13^, ^14^, ^15^ and it remains unclear whether PGT‐A increases the cumulative live birth rate per egg retrieval cycle or intention to treat.^16^ In addition, because PGT‐A, but not PGT‐SR, was prohibited in Japan for a long time, the effects of PGT‐A on infertile Japanese patients have not been elucidated.^17^

A previous pilot study conducted by the Japan Society of Obstetrics and Gynecology (JSOG) showed that PGT‐A improved the live birth rate per ET in patients that had experienced recurrent implantation failure (RIF) or recurrent pregnancy loss (RPL), but did not improve the live birth rate per patient or reduce the miscarriage rate.^18^ However, it is possible that the sample size of this pilot study was too small to detect a significant beneficial effect on the clinical miscarriage rate. Therefore, JSOG conducted a nationwide clinical study with a large study population to evaluate the pregnancy outcomes of PGT‐A/SR in patients that had experienced RIF or RPL or exhibited CR. Here, we summarize the data for 10 602 registered cycles collected in this clinical study.

## 2 MATERIALS AND METHODS

### 2.1 Study design

We conducted a multi‐center open‐label clinical trial, involving patients recruited from 200 fertility centers and testing at 17 laboratories in Japan. Each fertility center followed their own standard of care regarding ovarian stimulation, oocyte retrieval, IVF procedures, endometrial preparation, luteal‐phase support, and ET. In each case, a trophectoderm (TE) biopsy was performed on a good quality blastocyst from around five TE cells located apart from the inner cell mass, and the biopsy sample was then transferred to a genetic testing laboratory. After the TE biopsy, the blastocysts were vitrified.

The genetic testing laboratories were required to have established processes based on their own internally validated testing/reports and to meet known sequencing quality metrics. This study was approved by the research ethics committee of the JSOG and Tokushima University Hospital. In addition, appropriate approvals were obtained from institutional review boards or ethics committees in a site‐specific manner. Written informed consent was obtained from each couple before the procedures were performed.

Patients were recruited from December 1, 2019, to August 31, 2022. The clinical outcome follow‐up period ended on November 30, 2022. Patients with a history of two or more consecutive episodes of implantation failure after IVF‐ET treatment were enrolled as patients that had experienced RIF. The exclusion criteria for RIF were an abnormal chromosome in one or both partners or severe maternal complications. Patients with a history of two or more clinical miscarriages with or without IVF‐ET were enrolled as patients that had experienced RPL. The exclusion criteria for RPL were an abnormal chromosome in one or both partners, severe maternal complications, a congenital uterine anomaly, or antiphospholipid syndrome. In all cases involving RPL, both partners were subjected to chromosome analysis. Patients that exhibited CR during IVF‐ET were also enrolled regardless of whether they had a history of pregnancy and miscarriage. The only exclusion criterion for CR was severe complications. Enrollment was completed before oocyte retrieval, and the patients received no financial incentives for participation. All cycles were registered temporarily, and full registration was permitted after the study protocol had been completed. Cycles in which full registration did not occur within 6 months of temporary registration were regarded as dropouts.

### 2.2 Whole‐genome amplification and comprehensive chromosome screening

A whole‐genome amplification (WGA) and next‐generation sequencing (NGS)‐based assay or array comparative genomic hybridization was performed at each genetic testing laboratory, according to standard protocols and the manufacturers' recommendations. According to the results of the analysis, blastocysts were classified into the following four groups: euploid (A), euploid with suspected mosaicism (B), aneuploid (C), or undiagnosable (D). Blastocysts that demonstrated small variations, but could not be confirmed as aneuploid, were classified as exhibiting mosaicism. For patients in whom one or more blastocysts were classified into group A or B, a frozen–thawed single ET was performed. In cases in which no group A or B blastocysts were obtained, the ET was canceled. Information about the sex chromosomes of each embryo was not disclosed, except in cases with sex chromosome abnormalities.

### 2.3 Outcomes

The primary study outcome was the ongoing pregnancy rate at 12 weeks of gestation for each enrolled patient. The secondary study outcomes were the clinical pregnancy rate per ET and the miscarriages rate per clinical pregnancy. Cases in which a gestational sac formed were diagnosed as clinical pregnancies, and cases in which spontaneous or unplanned loss of a fetus from the uterus before 12 weeks of gestation were diagnosed as miscarriage. Cases with missing outcomes were excluded from analysis.

## 3 RESULTS

**FIGURE 1:**
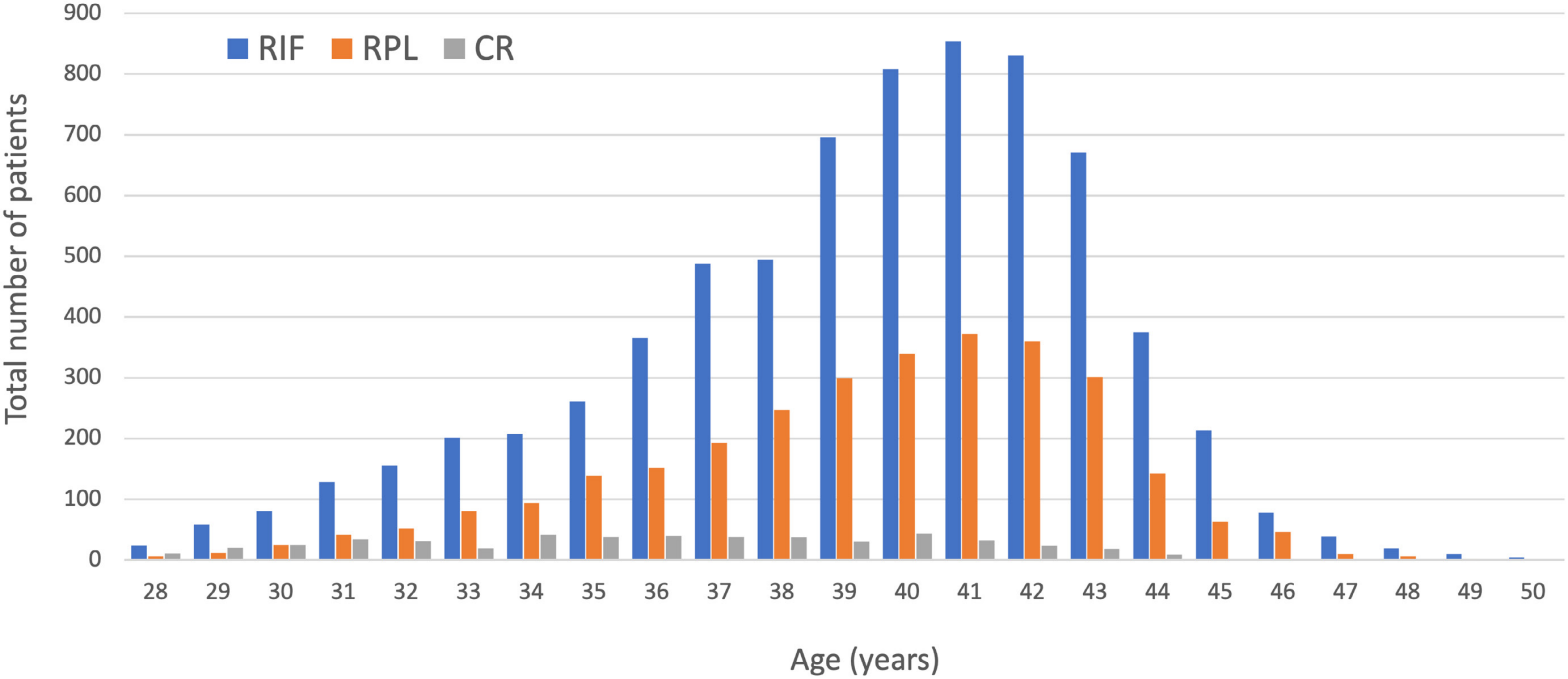
Distributions of maternal age among the patients with recurrent implantation failure (RIF), recurrent pregnancy loss (RPL), and chromosomal structural rearrangement (CR) in all registered cycles (*n* = 10 602).

During the study period, a total of 10 602 cycles (maternal age 28–50 years) were registered. The 7099, 2993, and 510 cycles were performed for RIF, RPL, and CR, respectively. The detailed characteristics and history of the patients in all registered cycles are shown in Table 1. The mean (SD) maternal age was 39.3 (3.9), and the mean (SD) number of prior pregnancies, live birth, and miscarriages were 1.5 (2.2), 0.26 (0.5), and 1.2 (1.3), respectively. The distributions of maternal age among the patients with RIF, RPL, and CR in all registered cycles are shown in Figure 1. The numbers of patients that had experienced RIF and RPL were highest at 41 years of age, and the number of patients with CR peaked at 40 years of age. 53.8% of the enrolled women were 40 years of age or older.

**TABLE 1:** Background characteristics of the patients in all registered cycles.

Variables	RIF (*n* = 7099)	RPL (*n* = 2993)	CR (*n* = 510)	Total (*n* = 10 602)
Age of female partner (years)	39.3 ± 4.0	39.4 ± 3.7	35.7 ± 4.6	39.3 ± 3.9
Age of male partner (years)	40.8 ± 5.9	40.6 ± 5.6	37.5 ± 6.0	40.8 ± 5.8
Duration of infertility (months)	44.2 ± 34.6	37.6 ± 40.7	34.9 ± 38.1	42.3 ± 36.7
Previous conception	0.9 ± 2.2	3.0 ± 1.2	2.3 ± 1.7	1.5 ± 2.2
Previous live birth	0.24 ± 0.48	0.31 ± 0.53	0.31 ± 0.50	0.26 ± 0.50
Previous miscarriage	0.6 ± 0.8	2.6 ± 1.1	1.9 ± 1.5	1.2 ± 1.3
Previous egg retrievals	3.9 ± 3.9 (*n* = 7099)	3.3 ± 4.7 (*n* = 2292)	1.7 ± 3.1 (*n* = 243)	3.7 ± 4.2 (*n* = 9634)
Previous ET cycles	4.8 ± 3.3 (*n* = 7099)	3.4 ± 3.4 (*n* = 2292)	1.9 ± 3.1 (*n* = 243)	4.4 ± 3.4 (*n* = 9634)

*Note*: Data are expressed as mean ± SD.

Abbreviations: CR, chromosomal structural rearrangement; ET, embryo transfer; *n*, total number of cycles; RIF, recurrent implantation failure; RPF, recurrent pregnancy loss.

**FIGURE 2:**
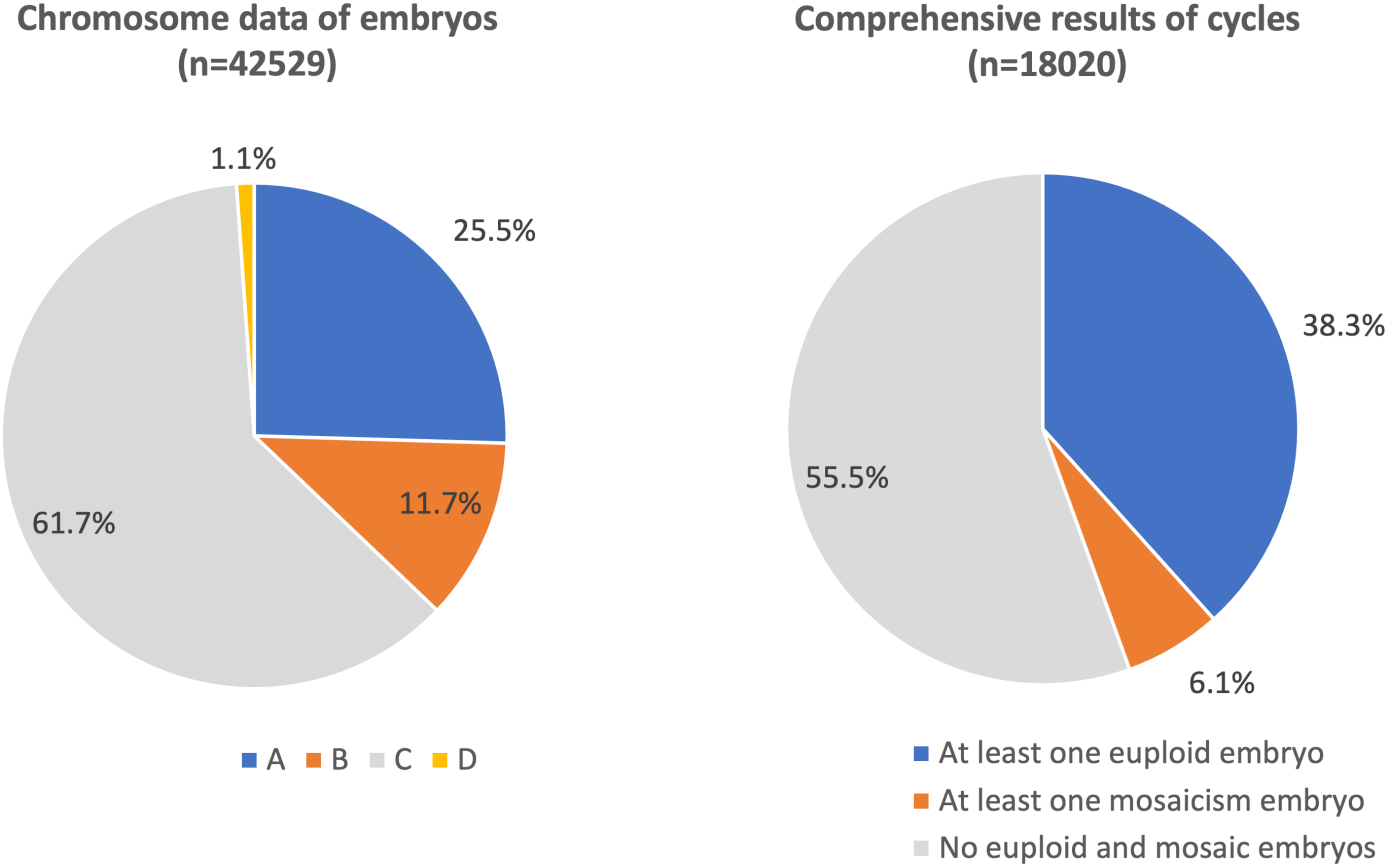
Distribution of euploidy (A), mosaicism (B), aneuploidy (C), and undiagnosable blastocysts (D) among the blastocysts and comprehensive results. Data in all categories (RIF, RPL, and CR) are included. CR, chromosomal structural rearrangement; RIF, recurrent implantation failure; RPL, recurrent pregnancy loss.

**FIGURE 3:**
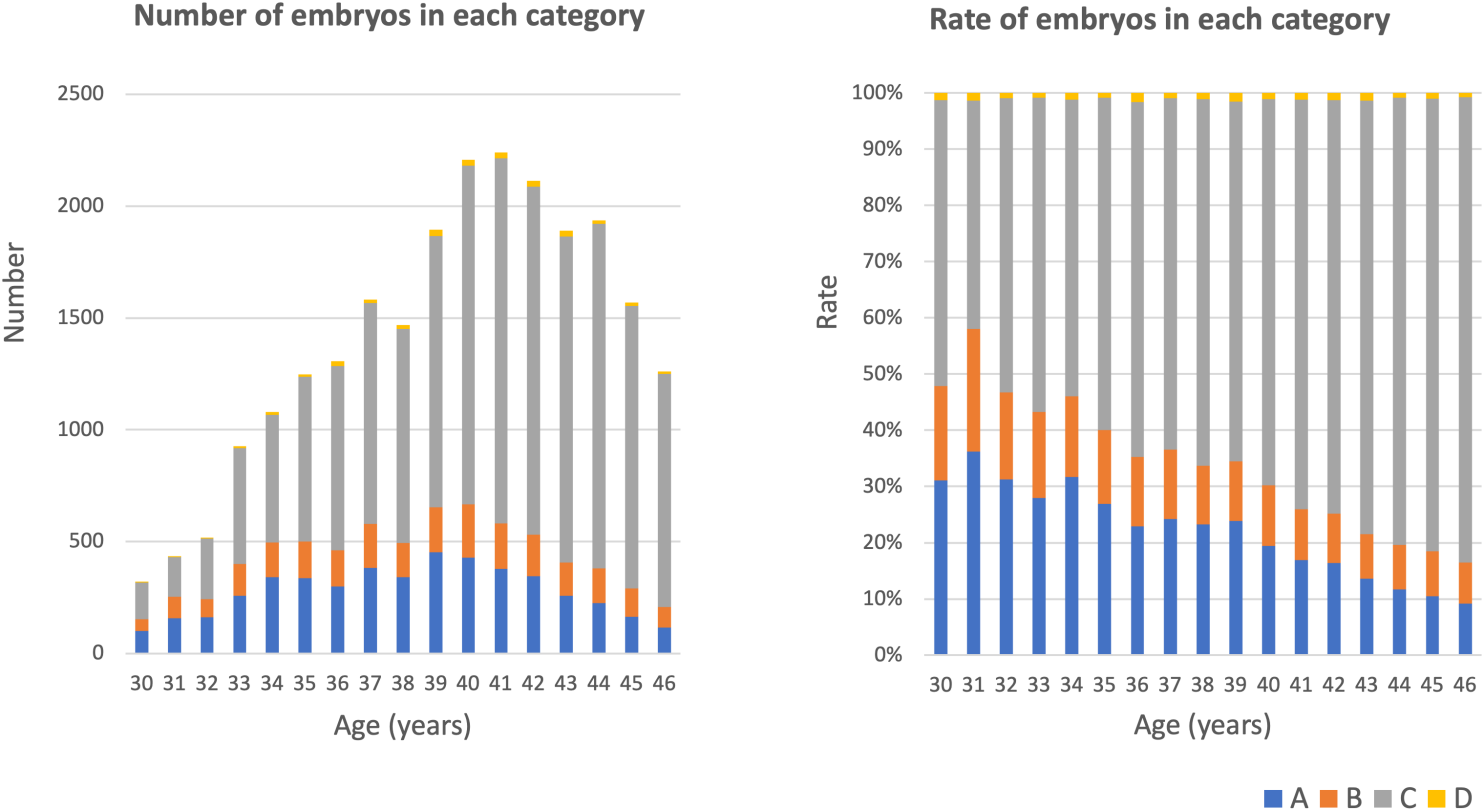
Numbers and rates of euploidy (A), mosaicism (B), aneuploidy (C), and undiagnosable blastocysts (D), stratified by maternal age. Data of embryos in all categories (RIF, RPL, and CR) are included. CR, chromosomal structural rearrangement; RIF, recurrent implantation failure; RPL, recurrent pregnancy loss.

**FIGURE 4:**
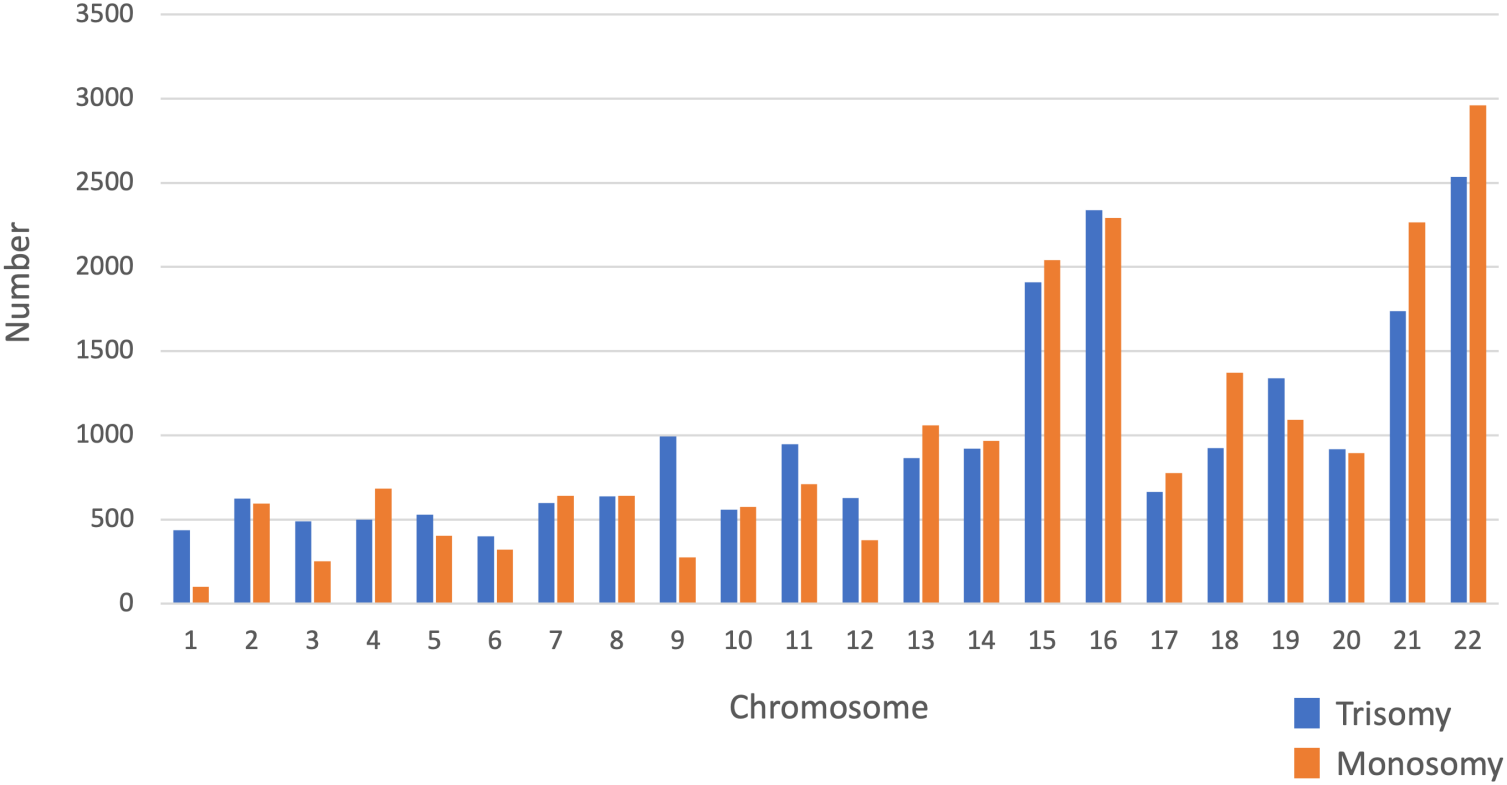
Numbers of autosomal trisomy and monosomy in each chromosome. Data in all categories (RIF, RPL, and CR) are included. A total of 46 404 abnormalities are detected. CR, chromosomal structural rearrangement; RIF, recurrent implantation failure; RPL, recurrent pregnancy loss.

A total of 42 529 blastocysts were biopsied for PGT‐A/SR. The distribution of euploidy, mosaicism, aneuploidy, and undiagnosable blastocysts among the blastocysts from all patients (RIF, RPL, and CR) is shown in Figure 2. 25.5% of the embryos were euploid (A), 11.7% demonstrated mosaicism (B), 61.7% were aneuploid (C), and only 1.1% were undiagnosable (D). At least one euploid blastocyst was obtained in 38.3% of egg retrieval cycles with embryo biopsy, whereas only aneuploid and/or mosaic embryos, but not euploid blastocysts, were obtained in 61.7% of egg retrieval cycles with embryo biopsy. The numbers and rates of euploidy (A), mosaicism (B), aneuploidy (C), and undiagnosable blastocysts (D), stratified by maternal age, among all patients (RIF, RPL, and CR) are shown in Figure 3. The proportion of aneuploid embryos increased with maternal age, from 30.0% in women of 30–35 years of age to 14.5% in women of 40–46 years of age. The frequency of aneuploidy for each chromosome among the embryos from all patients (RIF, RPL, and CR) is shown in Figure 4. A total of 46 404 chromosomal abnormalities were detected in the aneuploid embryos, with monosomy and trisomy equally represented.

**FIGURE 5:**
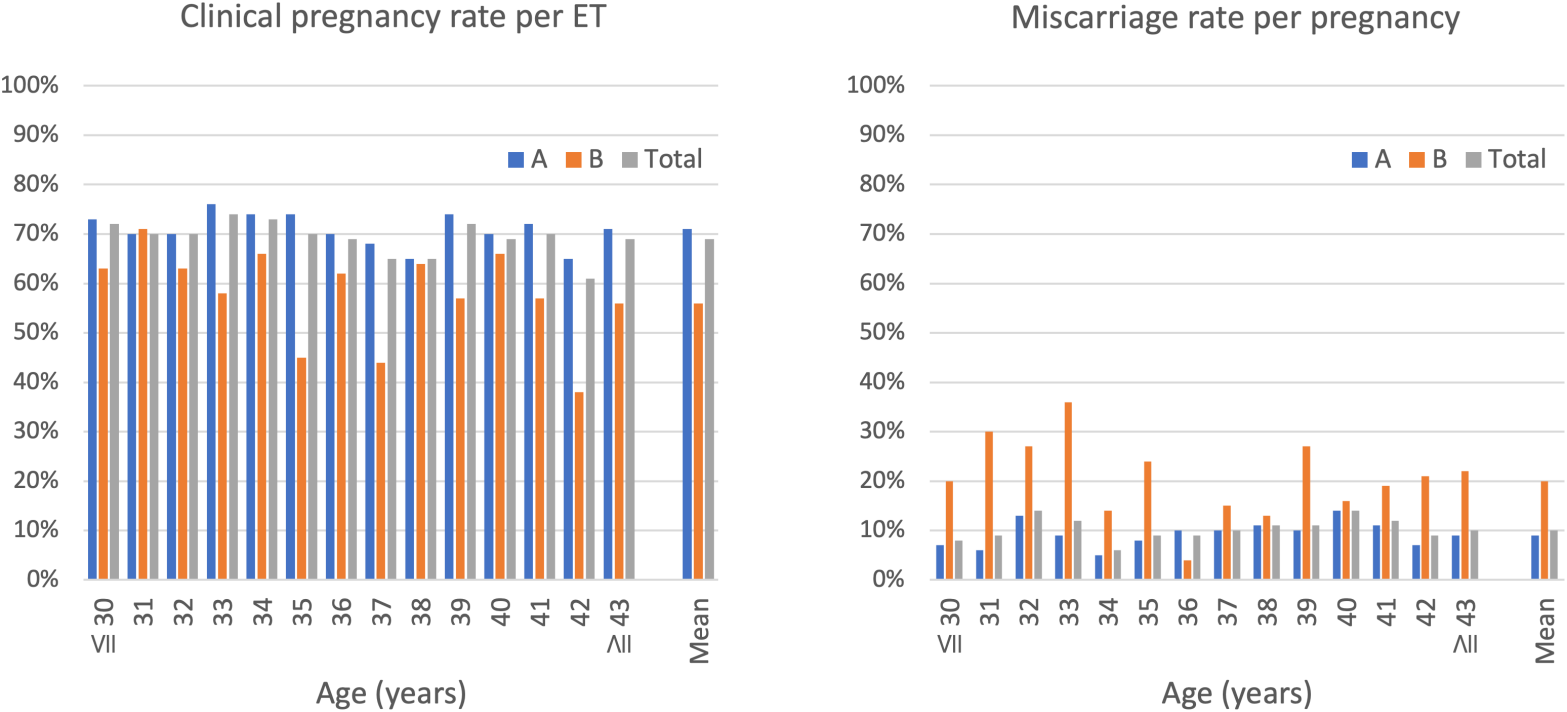
Rates of clinical pregnancy and miscarriage stratified by maternal age in euploid (A) and mosaic (B) embryo transfer. Data in all categories (RIF, RPL, and CR) are included. CR, chromosomal structural rearrangement; RIF, recurrent implantation failure; RPL, recurrent pregnancy loss.

A total of 6080 ETs were carried out, and the clinical outcomes of the transferred embryos are shown in Table 2. Outcomes were missed in 1419 ETs and 344 pregnancies. The clinical pregnancy rate per ET and miscarriage rate per pregnancy were 65.5% and 9.9% in the cases involving RIF, 74.7% and 11.1% in the cases involving RPL, and 80.7% and 11.8% in the cases involving CR, respectively. Ongoing pregnancy rate per ET were 53.9% in the cases involving RIF, 60.7% in the cases involving RPL, and 66.1% in the cases involving CR, respectively. The clinical pregnancy rate per ET and miscarriage rate per pregnancy, stratified by maternal age, among all patients (RIF, RPL, and CR) are shown in Figure 5. The rates of clinical pregnancy and miscarriage remained relatively constant across all maternal ages. In addition, in mosaic embryo (B) transfer, implantation rate is low and miscarriage rate is high compared with those in euploid embryo (A) transfer.

**TABLE 2:** Clinical outcomes.

Variables	RIF	RPL	CR	Total
Oocyte retrieval cycles, *n*	16 136	7650	1283	25 069
PGT‐A/SR cycles, *n*	11 626	5282	1019	17 927
ET cycles, *n*	4101	1663	316	6080
Clinical pregnancies, *n*	2688	1243	255	4186
Clinical pregnancy rate per ET, %	65.5%	74.7%	80.7%	68.8%
Ongoing pregnancy, *n*	2209	1009	209	3425
Ongoing pregnancy rate per ET, %	53.9%	60.7%	66.1%	56.3%
Miscarriage, *n*	244	126	28	398
Miscarriage rate per pregnancy, %	9.9%	11.1%	11.8%	10.4%
Ectopic pregnancies, *n*	12	6	1	19
Outcome unknown, *n*	223	104	17	344

*Note*: Cases with missing outcomes were excluded from analysis.

Abbreviations: CR, chromosomal structural rearrangement; ET, embryo transfer; RIF, recurrent implantation failure; RPF, recurrent pregnancy loss.

## 4 DISCUSSION

In this study, we summarized the data collected in a nationwide clinical study of PGT‐A/SR conducted by JSOG. During the study period, 10 602 cycles were registered, 42 529 blastocysts were biopsied for PGT‐A/SR, and 6080 ETs were carried out. As expected, RIF was the most common indication for PGT‐A/SR, and it accounted for 70.0% of the cases. Regarding the distribution of maternal age, the mean age of the patients that underwent PGT‐A/SR was 39.1 years of age; a little older than the mean age (37.8 years of age) of the patients that underwent ART in Japan in 2020.^5^ As around 90% of patients had undergone several cycles of ART, and some of them had experienced two or more miscarriages before participating in this study, the higher maternal age of the patients that underwent PGT‐A/SR in this study may have been due to their longer treatment history.

In total, the euploidy rate was 25.5% in this study, and at least one euploid blastocyst was obtained in 38.3% of egg retrieval cycles. These results are similar to those obtained in our pilot study,^18^ whereas they are much lower than those seen in previous studies performed in other countries.^4^, ^19^ Euploid blastocysts and euploid blastocysts with suspected mosaicism were classified into separate categories in this study, whereas these two groups were simply categorized as euploid blastocysts in previous studies and the criteria for designating mosaicism differ among centers.^20^ These differences in definitions may explain the discrepancies in the abovementioned results. The proportion of euploid embryos decreased after 37 years of age and reached <15% at 43 years of age or older. This tendency is similar to those seen in previous studies, for example, the euploid rate was highest between the ages of 26 and 30 and steadily decreased through to age 43 and then plateaued.^4^ Taken together, these findings indicate that around 40% of patients may produce at least one euploid blastocyst per egg retrieval cycle and that the euploid blastocyst per egg retrieval cycle rate decreases as maternal age rises. In other words, about 60–70% of patients may not have any euploid blastocysts after PGT‐A/SR, resulting in ET being canceled in these egg retrieval cycles. Aneuploidies were detected in all chromosomes in this study, and high frequencies of aneuploidy were seen in chromosomes 15, 16, 21, and 22. These trends corresponded to the results of previous studies of the chromosomal abnormalities seen in PGT‐A^21^ and the products of conception collected after miscarriages.^22^

In this study, the clinical pregnancy rate per ET was about 68.8%, and the miscarriage rate per pregnancy was about 10.4%, and these pregnancy and miscarriage rates continued to be seen at advanced maternal age. In contrast, it has been reported that the pregnancy rate per ET was 33.9% and the miscarriage rate per pregnancy was 24.9% in patients that underwent ART in Japan in 2020 and that these rates worsened with age.^5^ Thus, as described in previous studies, pregnancy outcomes per ET may be improved by PGT‐A/SR in the infertile populations examined in the current study, especially in patients of advanced maternal age. Although clinical pregnancy rate was lower and miscarriage rate was higher in mosaic embryo transfer than in euploid embryo transfer, this clinical pregnancy rate of mosaic embryo transfer might still better than that collected from ART registry system.^5^ Anyway, more data about mosaic embryo transfer should be accumulated and its safety and efficacy should be discussed in the future.

This study had some limitations. First, because there were no controls; that is, a similar infertile population that did not undergo PGT‐A/SR, the efficacy of PGT‐A/SR could not be precisely evaluated. Second, because combined data for RIF, RPL, and CR were used for most of the analyses, the efficacy of PGT‐A/SR in each group could not be evaluated. Third, although pregnancy outcomes per ET or pregnancy were evaluated, outcomes per patient, such as the pregnancy rate per patient and cumulative live birth rate per egg retrieval cycle or intention to treat, could not be examined.

To conclude, a nationwide clinical study showed that PGT‐A/SR may improve the pregnancy rate per ET and reduce the miscarriage rate per pregnancy in cases involving RIF, RPL, or CR, especially in patients of advanced maternal age. On the contrary, euploid blastocysts cannot be obtained in 60% or more of egg retrieval cycles, and it remains unclear whether PGT‐A/SR can improve the cumulative live birth rate per egg retrieval cycle or intention to treat.

## CONFLICT OF INTEREST STATEMENT

The authors declare no conflict of interest.

## ETHICS STATEMENT

This study was approved by the research ethics committee of the JSOG and Tokushima University Hospital. In addition, site‐specific appropriate institutional review board or ethics committee approval was obtained.

## HUMAN RIGHTS STATEMENTS AND INFORMED CONSENT

All procedures were performed in accordance with the ethical standards of the relevant committees on human experimentation (institutional and national) and the Helsinki Declaration of 1964 and its later amendments.

## ANIMAL RIGHTS

This report does not contain any studies performed by any authors that included animals.
