# Correction to “Preimplantation Genetic Testing for Aneuploidy and Chromosomal Structural Rearrangement: A Summary of a Nationwide Study by the Japan Society of Obstetrics and Gynecology”

**DOI:** 10.1002/rmb2.12676

**Published:** 2025-09-08

T. Iwasa, A. Kuwahara, T. Takeshita, Y. Taniguchi, M. Mikami, M. Irahara, “Preimplantation Genetic Testing for Aneuploidy and Chromosomal Structural Rearrangement: A Summary of a Nationwide Study by the Japan Society of Obstetrics and Gynecology,” *Reproductive Medicine and Biology* 22 (2023): e12518, https://doi.org/10.1002/rmb2.12518.

In Figure 3, the numbers and rates of euploidy (A), mosaicism (B), aneuploidy (C), and undiagnosable blastocysts (D) were originally shown as stratified by maternal age; however, they were actually plotted based on paternal age. The corrected figure, stratified by maternal age, is provided below. This correction does not affect the direction of the discussion or the conclusions.

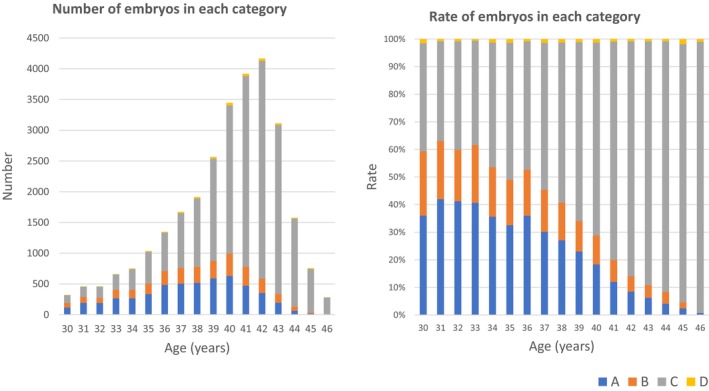

Due to the correction of Figure 3, the following sentence in the RESULTS section was published incorrectly:

The proportion of aneuploid embryos increased with maternal age, from 30.0% in women of 30–35 years of age to 14.5% in women of 40–46 years of age.

The correct sentence is:

The proportion of euploid embryos decreased with maternal age, from 30%–40% in women of 30–35 years of age to 20% or below in women of 40–46 years of age.

We apologize for these errors.

